# Hypertensive disorders of pregnancy, maternal cardiovascular disease mortality and the role of familial predisposition: a Norwegian population-based sibling-comparison, sibling-spillover and negative-control cohort study

**DOI:** 10.1093/aje/kwaf257

**Published:** 2025-11-17

**Authors:** Aditi Singh, Sage Wyatt, Liv Grimstvedt Kvalvik, Rolv Skjærven

**Affiliations:** Department of Global Public Health and Primary Care, University of Bergen, Bergen, Norway; Department of Global Public Health and Primary Care, University of Bergen, Bergen, Norway; Department of Global Public Health and Primary Care, University of Bergen, Bergen, Norway; Department of Global Public Health and Primary Care, University of Bergen, Bergen, Norway; Center for Fertility and Health, Norwegian Institute of Public Health, Oslo, Norway

**Keywords:** hypertensive disorders of pregnancy, pre-eclampsia, gestational hypertension, cardiovascular disease mortality, sibling-comparison, sibling-spillover, negative-control, familial predisposition

## Abstract

Hypertensive disorders of pregnancy (HDP) are associated with increased maternal cardiovascular disease (CVD) mortality, with risks varying by HDP subtypes and subsequent pregnancy outcomes. The contribution of shared familial factors given this heterogeneity is unclear. We conducted a population-based study using Norwegian registries (1967-2020) including 1 106 658 women with complete pregnancy histories, of whom 628 345 had at least one full sibling. Women with HDP were classified into low-risk (gestational hypertension or term pre-eclampsia followed by no HDPs) and high-risk (all other patterns) trajectories. CVD mortality before age 70 was assessed using population-level and sibling-based models: sibling-comparison (discordant-sisters), sibling-spillover (by sister’s HDP history), and negative-control models (by sister-in-law’s HDP history). CVD mortality among women with HDP varied by trajectory (population-level adjusted hazard ratios [aHR]_low-risk_ 1.03 [95% confidence intervals, 0.89-1.20]; aHR_high-risk_ 1.89 [1.74-2.06]). These differences persisted when compared to sisters without HDP (sibling-comparison aHR_low-risk_ 0.66 [0.44-1.01]; aHR_high-risk_ 1.51 [1.16-1.97]). Women without HDP had slightly elevated CVD mortality if sisters had HDP (sibling-spillover aHR_low-risk_ 1.28 [1.03-1.60]; aHR_high-risk_ 1.25 [1.06-1.49]), but not if sisters-in-law had HDP (negative-control aHR_low-risk_ 1.10 [0.85-1.40]; aHR_high-risk_ 1.01 [0.83-1.22]). Individual-specific factors drive the CVD mortality heterogeneity among women with HDP. Shared familial factors modestly elevate CVD mortality in women without HDP.

## Introduction

Women who experience hypertensive disorders of pregnancy (HDP) face increased risks of long-term cardiovascular disease (CVD) morbidity^1^^-^^11^ and mortality.^11^^-^^15^ The HDP-CVD association is increasingly recognized as heterogeneous,^9^^-^^14^ varying by HDP subtypes (gestational hypertension [GH], early-onset and late-onset pre-eclampsia [PE]) and subsequent pregnancy outcomes (no further pregnancies after the first HDP or HDP occurrence in subsequent pregnancies). For instance, women with recurrent HDP, preterm PE, HDP in later pregnancies, or those who do not pursue additional pregnancies following their first HDP (“high-risk” trajectories) have substantially higher CVD risks than those with term PE or GH followed by no HDP in subsequent pregnancies (“low-risk” trajectories). While the underlying mechanisms remain unclear,^16^^-^^19^ this heterogeneity is posited to arise from pathways involving placental dysfunction.^20^^-^^27^ These pathways include, for instance, shared risk factors predisposing to both HDP and CVD, as well as downstream effects of HDP through inflammation and endothelial dysfunction. Each pregnancy acts as a cardiovascular “stress test” thereby providing insights into long-term CVD risk stratification and revealing whether cardiovascular dysfunction is transient or progressive.^17^^,^^28^

Sibling-based designs^29^ can distinguish between factors shared between siblings that increase susceptibility to both HDP and CVD (hereafter referred to as shared familial factors) and individual-specific factors such as downstream effects of HDP or unique susceptibility factors that increase the risk of both HDP and CVD. These designs utilize sisters, who share approximately 50% of their genetic makeup and similar early-life environments, as well as sisters-in-law, who share similar adult environments but not genetics. Previous sibling-based studies have reported conflicting findings regarding the contribution of shared familial factors in the HDP-CVD association.^30^^-^^36^ Moreover, most were limited to HDP in the first pregnancy or did not account for the order of HDP-affected pregnancies. To our knowledge, no prior study has examined whether shared familial factors can explain the observed heterogeneity in the HDP-CVD mortality association.

In this study, we assess how shared familial factors contribute to the HDP-CVD mortality, accounting for heterogeneity using trajectories based on complete pregnancy history (no HDP, low-risk HDP, and high-risk HDP). We employ four analytical approaches: (1) population-level models, comparing CVD mortality among women with and without HDP in the population; (2) sibling-comparison models, assessing the role of individual-specific factors by controlling for shared familial factors through comparisons of CVD mortality among sisters where one has HDP and the other does not; (3) sibling-spillover models, evaluating the role of shared familial factors by examining CVD mortality among women without HDP according to their sister’s HDP status; and (4) negative-control models, assessing residual confounding by examining CVD mortality among women without HDP based on their sister-in-law’s HDP status. Specifically, we address two questions: (1) Does familial predisposition to HDP increase CVD mortality in women who personally do not develop HDP? (2) Do women with HDP have similar CVD mortality as their sisters who do not develop HDP?

## Methods

### Data sources

We linked data from Norwegian population-based registries using pseudo-anonymized personal identification numbers. The Medical Birth Registry of Norway (MBRN), which has mandated reporting of all pregnancies from gestational week 16 since 1967, provided data on pregnancy complications, fetal outcomes, maternal pre-pregnancy comorbidities, and demographics.^37^ The National Population Register provided demographics and emigration data; parental information was available for individuals whose parents were alive during the 1960 Census or were Norwegian residents from 1964 onward.^38^ The Norwegian Cause of Death Registry provided data on underlying and contributing causes of death.^39^ Educational attainment data for Norwegian residents in 2019 were obtained from the National Educational Database.^40^

### Study population

**Figure 1:**
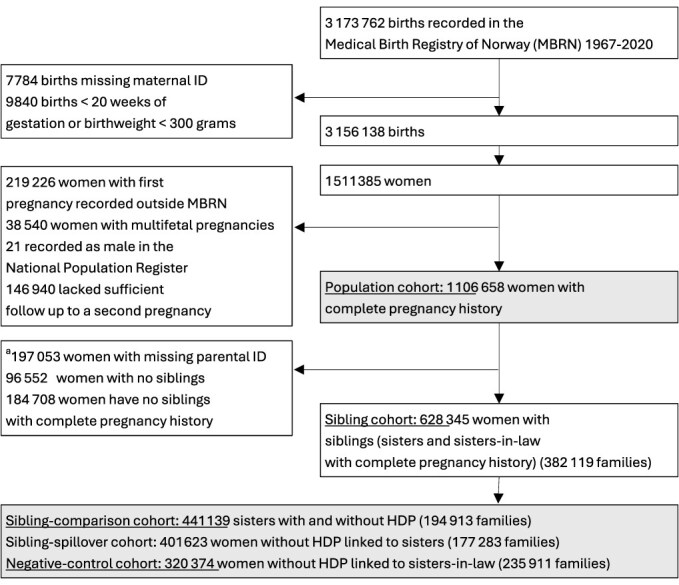
Flowchart of the study population. Norway, 1967-2020. Sister-in-law were identified as those who had all their pregnancies with only one partner (brother of the index woman). Abbreviation: MBRN medical birth registry of Norway; HDP, hypertensive disorders of pregnancy. ^a^exclusion based on age at first pregnancy, reflecting 90% of women that typically had a second pregnancy within: 9 years for ages below 20; 7 years for ages 20-29; 5 years for ages 30-39 and 4 years for ages ≥40.

We identified 1 511 085 women with 3 156 138 pregnancies (≥20 weeks’ gestation or birthweight ≥300 grams) registered between 1967 and 2020. We ascertained complete pregnancy histories for 1 106 658 women, of whom 628 345 had at least one full sister or sister-in-law with complete pregnancy histories (Figure 1).

### Cardiovascular disease mortality

The main outcome was CVD death before age 70, defined as death from ischemic heart disease (International Statistical Classification of Diseases and Related Health Problems [ICD]-8 and ICD-9 codes 410-414; ICD-10 codes I20-I25), cerebrovascular disease (ICD-8 and ICD-9 codes 430-438; ICD-10 codes I60-I69) and peripheral arterial disease (ICD-8 and ICD-9 code 443.9; ICD-10 code I73.9), recorded as either the underlying or a contributing cause of death.

### Hypertensive disorders of pregnancy

GH was defined as new-onset hypertension (systolic blood pressure ≥ 140 mmHg or diastolic blood pressure ≥ 90 mmHg) after 20 weeks of gestation. PE was defined as GH combined with proteinuria (≥0.3 grams/24 h urine or ≥ 1+ on dipstick). Cases of chronic hypertension-superimposed PE, eclampsia, and hemolysis, elevated liver enzymes, and low platelets (HELLP) syndrome were also classified as PE. Before 1999, PE and GH were recorded as free text in the MBRN, with PE diagnosis based on at least one measurement each of hypertension and proteinuria. From 1999 onward, reporting shifted to prespecified checkboxes, with PE diagnosis requiring two separate measurements of hypertension and proteinuria. Preterm PE was defined as delivery <37 weeks of gestation. For first pregnancies missing gestational age (5.3%), preterm status was assigned if birthweight was <2300 grams, which corresponds to the 10th percentile birthweight for gestational week 36.^41^ Validation studies indicate acceptable registration of HDP, with a positive predictive value of 88% and specificity of 99%; most misclassification involved underreporting of milder HDP cases, especially before 1999.^42^^-^^46^

Women with HDP were categorized as “low-risk” (GH or term PE in the first pregnancy followed by no HDP in subsequent pregnancies) or “high-risk” (comprising all other patterns of HDP, including HDP in first pregnancy and no further pregnancies, HDP in later pregnancies only, HDP in both first and later pregnancies and preterm PE in any recorded pregnancy). These risk categories were informed by population-level risk estimates (Supplementary Figure S1) following previous frameworks.^11^^,^^12^^,^^14^ For comparison, we also analyzed women with HDP without applying this categorization.

### Statistical analysis

We used Cox proportional hazards models to estimate hazard ratios (HRs) and 95% confidence intervals (CIs) for CVD mortality. Attained age was used as the underlying time scale. Follow-up for each woman began at the time of her last pregnancy and continued until death, emigration, or the end of the study period (December 31, 2020), whichever occurred first. Survival times were right censored at age 70. All analyses were performed using STATA version 18.5 and R version 4.4.2.

**Figure 2:**
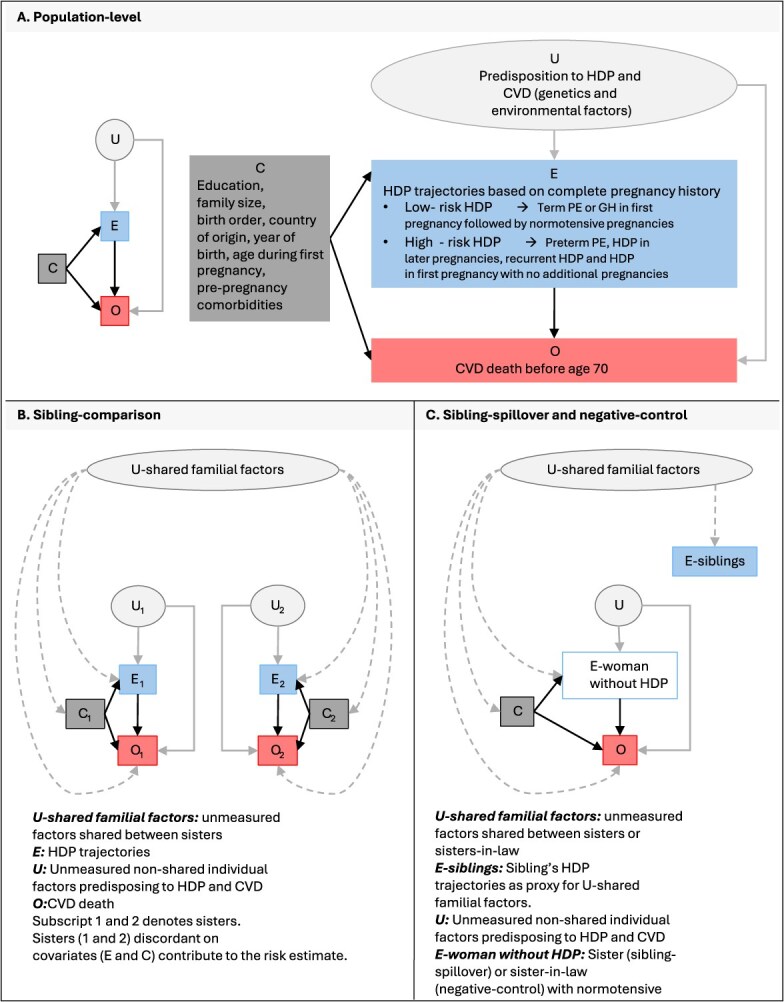
Directed acyclic graph illustrating the analytical workflow for the population-level and sibling-based models. Rectangles represent observed variables, while unobserved variables are represented with rounded corners. Dashed arrows indicate the direction of the association between various covariates and unmeasured shared familial factors. Abbreviations: CVD, cardiovascular disease; HDP, hypertensive disorders of pregnancy.

We used four analytical approaches (Figure 2):

1. Population-level model: We compared CVD mortality among women with HDP to those without HDP in the population.
2. Sibling-comparison model: We compared CVD mortality among sisters with and without HDP using stratified Cox regression with family-specific baseline hazards (strata defined by parental identification number), which controlled for measured and unmeasured shared factors and isolated the HDP-CVD association attributable to individual-specific factors. Only families where two or more sisters differed in HDP status (or covariates in the adjusted models) contributed to the risk estimates.^47^
3. Sibling-spillover model: We compared CVD mortality among women without HDP according to their sister’s HDP status to assess the influence of shared familial factors. Women without HDP were categorized as “high-risk” if any sister had high-risk HDP and as “low-risk” if all sisters with HDP had low-risk trajectories.
4. Negative-control model: We estimated CVD mortality among women without HDP based on their sister-in-law’s HDP status to assess residual confounding, as sisters-in-law do not share genetic or early-life environments. Women without HDP were categorized as “high-risk” if any sister-in-law had high-risk HDP and as “low-risk” if all sisters-in-law with HDP had low-risk trajectories.

Models were adjusted for women’s year of birth, country of origin, age at first pregnancy, year of first pregnancy, pre-pregnancy comorbidities, total number of pregnancies and education, with additional adjustment for birth order and number of siblings in sibling-based models. Adjusted models used complete-case data (missing data for covariates country of origin <0.1% and education <3% across models).

### Sensitivity and subgroup analyses

To evaluate the robustness of our findings, we conducted a series of sensitivity and subgroup analyses.

We analyzed GH and PE separately to compare findings for binary and trajectory-based categorization with existing literature. Similar to the HDP risk trajectory approach, PE and GH were categorized as low-risk and high-risk groups. Women who experienced PE in one pregnancy and GH in another pregnancy (regardless of sequence) were included in both the high-risk GH and high-risk PE categories.

To quantify the contribution of shared familial factors, we compared CVD mortality between women without HDP categorized according to their sisters’ HDP status and women with HDP of the corresponding trajectory.

To assess the specificity of the HDP-CVD mortality association, we examined non-CVD mortality before age 70. Given the longer life expectancy of Norwegian women, we also extended follow-up to include CVD mortality after age 70.

To minimize bias from non-shared environmental factors, we restricted sibling-based analyses to siblings born within seven years of each other. For sibling-comparison models, sibling-matched cohorts were constructed by matching (with replacement) sisters with HDP to sisters without HDP born within seven years, with stratified Cox regression based on matched set ID.^48^^,^^49^ For sibling-spillover and negative-control models, exposure was defined based on siblings born within seven years of the index woman without HDP.

To assess the influence of reference groups with differing baseline CVD risk profiles in sibling-based models, we considered two alternative reference groups. First, we expanded the reference group to include women without children alongside women without HDP. Women without children were defined as Norwegian women born between 1930 and 1980 who survived to age 40 and had no pregnancies registered in the MBRN or offspring in the National Population Register (Supplementary Figure S2). Previous studies have shown that women without children exhibit higher CVD mortality.^50^^,^^51^ Second, we narrowed the reference group to women with no pregnancy complications, since sibling history of HDP is associated with an increased risk of placental dysfunction-related pregnancy complications linked to elevated CVD mortality.^52^^,^^53^

To address under-reporting of mild HDP, we performed record-level probabilistic bias analyses using Monte Carlo simulation (Supplementary Appendix S1; code adapted from Fox et al^54^ and bias parameters derived from Moth et al.^45^).

## Results

### Study population characteristics

Among the 1 106 658 women with complete pregnancy histories, 628 345 (57%) had at least one full sibling with a complete pregnancy history. Compared to women without siblings, those with siblings were more likely to be born between 1955 and 1974 (61% of women with siblings versus 32% of women without siblings). A larger proportion had Norwegian parents (95% versus 77%) and higher education ≥14 years (43% versus 40%). Women with siblings were also less likely to have only one pregnancy (15% versus 21%) and were younger at first pregnancy (≥30 years at first pregnancy: 18% versus 22%). Additionally, fewer women with siblings died before age 70 (3.6% versus 5.1%) (Supplementary Table S1).

Of the 96 241 women with HDP in the population cohort, 59 928 (62%) had high-risk trajectories and 36 313 (38%) had low-risk trajectories (Supplementary Table S2). Women with HDP had more pre-pregnancy comorbidities (ranging from 2.4% to 14% for high-risk, 1.8%-5.5% for low-risk versus 1.7% for no HDP). Regardless of HDP status, women with only one pregnancy were older at first pregnancy (mean age 29 versus 25 years) and more likely to have lower education < 11 years (21% versus 17-19%). Characteristics by HDP trajectory for the 39 516 women with HDP in the sibling cohort were comparable (Supplementary Table S3).

Of the 571 891 women without HDP, 251517 (44%) were linked to their sisters, 170 268 (30%) to their sisters-in-law, and 150 106 (26%) to both (Supplementary Table S4). Women without HDP whose sisters had HDP were more likely to have ≥2 sisters (45% for low-risk, 48% for high-risk versus 29% for no HDP), had a later birth order (70% for low-risk, 71% for high-risk versus 67% for no HDP) and had more pre-pregnancy comorbidities (1.9% for low-risk, 2.0% for high-risk versus 1.7% for no HDP) compared to those whose sisters had no HDP. Women without HDP whose sisters-in-law had HDP were more likely to have ≥2 sisters-in-law (40% for low-risk, 44% for high-risk versus 24% for no HDP) and had a later birth order (71% for low-risk, 72% for high-risk versus 69% for no HDP).

### Hypertensive disorders of pregnancy and cardiovascular disease mortality

**Figure 3:**
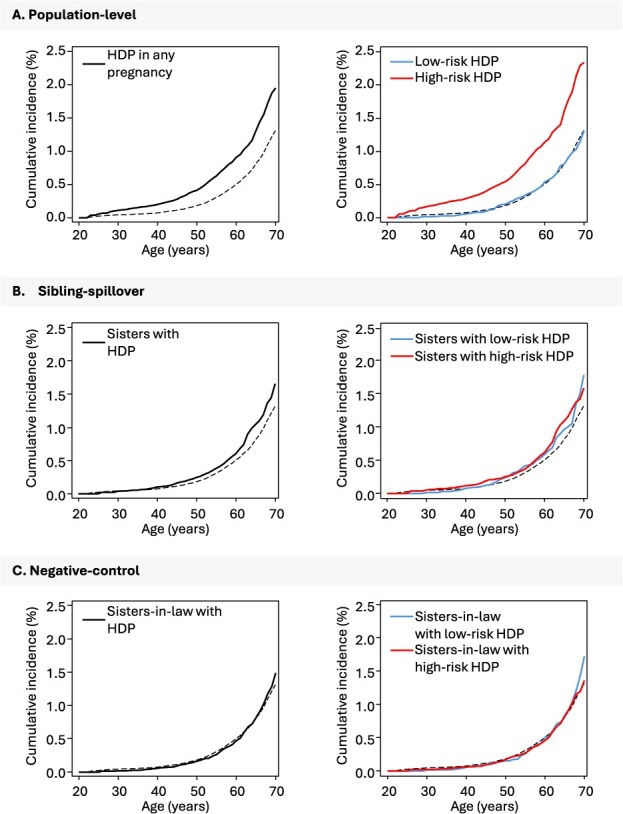
Cumulative risk of cardiovascular disease mortality before age 70 by hypertensive disorders of pregnancy. Norway, 1967-2020. Cumulative risks are calculated for women in the sibling-cohort. Panel A shows risk among women with HDP; panel B and C show risks among women without HDP according to their sibling’s HDP status (sisters in sibling-spillover models and sisters-in-law in negative-control models). The dashed black line overlaid in all panels represents risks in women from families without HDP. The solid lines denote cumulative risks based on HDP status: HDP in any pregnancy, low-risk HDP (term pre-eclampsia or gestational hypertension followed by no HDP in subsequent pregnancies) and high-risk HDP (all other patterns) trajectories. Abbreviation: HDP, hypertensive disorders of pregnancy.

**Figure 4:**
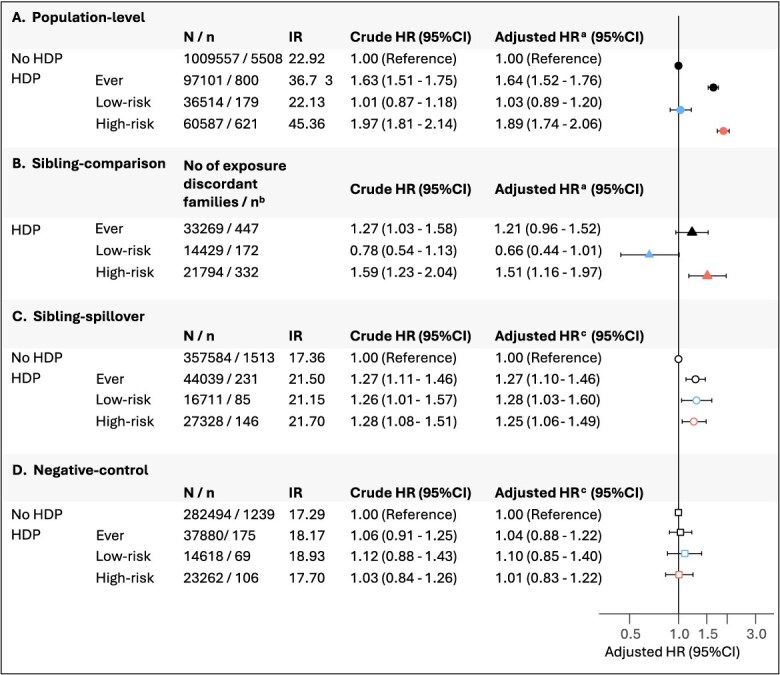
Hazard ratios for cardiovascular disease mortality before age 70 by hypertensive disorders of pregnancy. Norway, 1967-2020. HR are based on the population-cohort: Panel A shows HRs among women with HDP compared to those without HDP. Panel B, C and D are based on the sibling-cohort: Panel B shows HRs derived from sibling-comparison models; panel C and D compare CVD mortality in women without HDP according to their sibling’s HDP status (sisters in sibling-control models and sisters-in-law in negative-control models). Abbreviations: CI, confidence intervals; CVD, cardiovascular disease; HDP, hypertensive disorders of pregnancy; HR, hazard ratio; IR incidence rate per 100 000 person years. ^a^adjusted for women’s year of birth (restricted cubic splines with knots at 1955, 1965, and 1975), country of origin (Norwegian and other), age at first pregnancy (restricted cubic splines with knots at 20, 30, and 40 years), year of first pregnancy (restricted cubic splines with knots at 1975, 1985, and 1999), pre-pregnancy comorbidities (presence of chronic hypertension, chronic kidney disease, epilepsy, rheumatoid arthritis or pregestational diabetes mellitus: Yes and no), total number of pregnancies (1, 2, and ≥ 3), and highest educational attainment (<11, 11-13 and ≥ 14 years of schooling). ^b^number of exposure-discordant families where at least one sister developed the outcome (CVD death). ^c^Adjusted for covariates listed under (^a^), birth order (first-born and later-born), and number of siblings (1, 2, and ≥ 3; sisters for sibling-spillover model and sisters-in-law for negative-control model).

Figure 3 displays the cumulative incidence of CVD mortality in women with HDP and women without HDP according to their sister’s and sister-in-law’s HDP status. Figure 4 shows the adjusted hazard ratios (aHR) for CVD mortality before age 70 based on HDP status.

#### Population-level analysis

Women with HDP in any pregnancy had elevated CVD mortality with an aHR of 1.64 (95% CI, 1.52-1.76) compared to women without HDP. The association varied substantially by HDP trajectory. Women with low-risk HDP had an aHR of 1.03 (95% CI, 0.89-1.20), while those with high-risk HDP had an aHR of 1.89 (95% CI, 1.74-2.06).

#### Sibling-comparison analysis

Women with HDP in any pregnancy had an aHR of 1.21 (95% CI, 0.96-1.52) compared to their sisters without HDP. The trajectory-specific patterns observed in the population analysis were attenuated in the sibling-comparison model. Sisters with low-risk HDP had an aHR of 0.66 (95% CI, 0.44-1.01), while those with high-risk HDP had an aHR of 1.51 (95% CI, 1.16-1.97).

#### Sibling-spillover analysis

Compared to women without HDP whose sisters had no HDP, those whose sisters had HDP showed elevated CVD mortality with an aHR of 1.27 (95% CI, 1.10-1.46). Risks were similar regardless of HDP trajectory (aHR 1.28, 95% CI, 1.03-1.60 for low-risk HDP and aHR 1.25, 95% CI, 1.06-1.49 for high-risk HDP).

#### Negative control analysis

Women without HDP whose sisters-in-law had HDP showed no increased CVD mortality with an aHR of 1.04 (95% CI, 0.88-1.22) and showed no substantial difference between low-risk HDP (aHR 1.10, 95% CI, 0.85-1.40) and high-risk HDP (aHR 1.01, 95% CI, 0.83-1.22).

### Sensitivity and subgroup analyses

#### Separate analyses for PE and gestational hypertension

Separate PE and GH analyses showed similar patterns to the main findings involving HDP (Supplementary Figure S3). Women with PE or GH in any pregnancy had a 1.6 to 1.7-fold increased CVD risk (aHR 1.67, 95% CI, 1.52-1.82 for PE and aHR 1.61, 95% CI, 1.44-1.81 for GH). Women with high-risk trajectories had 1.8-1.9-fold higher risk (aHR 1.94, 95% CI, 1.76-2.15 for high-risk PE and aHR 1.76, 95% CI, 1.55-1.99 for high-risk GH) while those with low-risk trajectories had no increased risk compared to women without HDP (aHR 1.02, 95% CI, 0.85-1.23 for low-risk PE and aHR 1.06, 95% CI, 0.83-1.35 for low-risk GH).

Sibling-comparison models for PE produced results similar to those of the population-level models but were slightly attenuated. Sisters with PE in any pregnancy had an aHR of 1.55 (95% CI, 1.17-2.06), with an aHR of 0.97 (95% CI, 0.57-1.65) for low-risk PE and an aHR of 1.77 (95% CI, 1.28-2.44) for high-risk PE. Sisters with GH in any pregnancy had no increased CVD mortality risk compared to their sisters without HDP (aHR 0.83, 95% CI, 0.58-1.18). When categorized by trajectory, sisters with high-risk GH had CVD mortality risks similar to sisters without HDP (aHR 1.03, 95% CI, 0.68-1.55), while those with low-risk GH had 58% lower CVD mortality (aHR 0.42, 95% CI, 0.21-0.83).

In sibling-spillover models, women whose sisters had low-risk GH, high-risk GH or high-risk PE had a 1.2- to 1.5-fold increased risk, but no increased risk if sisters had low-risk PE (aHR 1.07, 95% CI, 0.80-1.44). Negative-control models showed that women without HDP had 58% higher CVD mortality only if their sisters-in-law had low-risk GH (aHR 1.58, 95% CI, 1.13-2.21).

#### Direct contribution of shared familial factors compared to HDP trajectories

Women without HDP whose sisters had low-risk HDP had an aHR of 1.27 (95% CI, 0.89-1.78) compared to women with low-risk HDP (Supplementary Table S5). Women without HDP whose sisters had high-risk HDP had an aHR of 0.67 (95% CI, 0.53-0.84) compared to women with high-risk HDP.

#### Other outcome endpoints, exposure definitions, and alternative reference groups

CVD mortality after age 70 was attenuated, though the heterogeneity persisted in population-level models (Supplementary Table S6). However, sibling-based models had fewer cases and less precise estimates with CIs that included the null.

For non-CVD mortality before age 70, no associations were observed. Sibling-comparison models suggested slightly lower CVD mortality risks for sisters with low-risk HDP (aHR 0.87, 95% CI, 0.74-1.03) and slightly higher risks for those with high-risk HDP (aHR 1.12, 95% CI, 0.99-1.26); however, the CIs included the null.

Restricting analyses to siblings born within seven years yielded similar findings to the main results, except in sibling-spillover models, where women without HDP who had sisters with high-risk HDP showed slightly lower risks (aHR 1.13, 95% CI, 0.92-1.39).

Alternative reference groups largely yielded similar findings. However, when using women without pregnancy complications as the reference in sibling-comparison models, the protective association for low-risk HDP was attenuated (aHR 0.82, 95% CI, 0.53-1.29). Including women without children in the reference group resulted in attenuation of the risk estimate for women without HDP but with sisters who had high-risk HDP (aHR 1.12, 95% CI, 0.95-1.33).

#### Probabilistic bias analysis to address mild HDP underreporting

The magnitude of bias depended on both the analytic model and misclassification scenario (Supplementary Table S7 and Supplementary Figure S4).

Four exposure misclassification scenarios were assessed: (scenario A) sampling of sensitivity, specificity and prevalence; (scenario B) direct sampling of positive and negative predictive values; (scenario C) scenario B plus additional misclassification probability if a younger sister had HDP; and (scenario D) scenario B plus reclassification of women with HDP and only one pregnancy as high-risk HDP. While population-level models showed minimal bias (0-2% for low-risk, 1% for high-risk in scenarios A-C, 38% in scenario D), greater bias was observed in sibling-comparison (-33% for low-risk, 2% for high-risk in scenarios A-C, 38% in scenario D) and sibling-spillover models (-33% for low-risk, 1%-2% for high-risk in scenarios A-C, 22% in scenario D).

## Discussion

### Key findings

In this population-based registry study, we employed population-level and sibling-based (sibling-comparison, sibling-spillover, and negative-control) models to investigate how shared familial factors contribute to the association between HDP and CVD mortality. Compared to women without HDP, those with high-risk HDP had a 1.9-fold higher CVD mortality risk, while those with low-risk HDP showed no increased CVD mortality risk. Compared to their sisters without HDP, women with high-risk HDP had a 1.5-fold higher CVD mortality risk, and those with low-risk HDP had a 0.7-fold lower CVD mortality risk. Women without HDP whose sisters had HDP exhibited a 1.3-fold increased CVD mortality risk, regardless of HDP trajectory. No such association was observed among sisters-in-law.

### Interpretation of findings

The differences in CVD mortality by HDP trajectories observed in population-level models persisted in sibling-comparison models. Compared to sisters without HDP, women with high-risk HDP had approximately 30% higher CVD mortality, while those with low-risk HDP showed no increased or slightly lower CVD mortality. High-risk HDP trajectories may reflect either underlying high-risk CVD profiles unmasked by the physiological demands of pregnancy or sustained cardiovascular damage caused by HDP-affected pregnancies, with both mechanisms operating through pathways involving placental dysfunction.^17^^-^^19^ Conversely, low-risk trajectories may suggest favorable cardiovascular profiles in which factors predisposing to HDP in the first pregnancy are less strongly linked to long-term CVD risk.^6^^-^^13^ Unexpectedly, women with low-risk HDP had lower CVD mortality than their unaffected sisters. Sensitivity analyses accounting for misclassification and alternative referents attenuated these apparent protective associations. Subgroup analyses showed this association was driven by low-risk GH. We also found elevated risks in unaffected sisters-in-law of women with low-risk GH. Thus, we hypothesize that this paradoxical protective association for low-risk HDP may be partly explained by residual confounding, exposure misclassification or cohort selection effects.

Sibling-spillover and negative-control models showed that unaffected sisters had modestly elevated CVD mortality, whereas unaffected sisters-in-law exhibited no such association. However, subtype-specific analyses revealed deviations: unaffected women showed no increased CVD mortality if their sisters had low-risk PE, but they had elevated CVD mortality if their sisters-in-law had low-risk GH. Based on these subtype-specific findings, we hypothesize that risk factors for term PE are less closely tied to familial aggregation of CVD risk factors, as sisters with low-risk PE had CVD mortality similar to that of sisters without HDP. Conversely, women with low-risk GH may have a more favorable CVD profile compared to their sisters without HDP, who may carry a higher burden of CVD risk factors. We note that these interpretations may be speculative due to the methodological concerns about low-risk GH as raised earlier. Although prior studies suggest poorer cardiometabolic profiles among unaffected siblings of individuals exposed to HDP in-utero^55^^,^^56^ and genetic studies identify pleiotropic variants linking HDP and CVD risk,^30^^,^^57^^-^^59^ none have investigated whether CVD risk factors among relatives of women with HDP vary based on the subtype- and trajectory-specific categories.

### Comparison with literature

Of the three studies using sibling-comparison models,^31^^-^^33^ two examined HDPs separately and found moderate to strong attenuations for HDPs in relation to ischemic heart disease^31^ and premature mortality.^32^ Replicating this approach, we found sibling-comparison model HRs were partially attenuated for PE and completely attenuated for GH compared to population-level model HRs. This suggests that CVD mortality associated with PE is predominantly driven by individual-specific factors, whereas CVD mortality associated with GH is primarily driven by shared familial factors. However, our trajectory-based analyses showed that only women with high-risk PE had elevated CVD mortality. Women with low-risk PE and high-risk GH had similar risks to their unaffected sisters, while low-risk GH was associated with lower CVD mortality. Thus, binary classification of HDP masks heterogeneity and may lead to oversimplified conclusions regarding the contribution of shared familial factors.

Findings on how a sibling’s HDP status influences CVD outcomes in women without HDP are inconsistent across studies due to methodological differences.^33^^-^^36^ Although our results align with more recent studies, these discrepancies likely arise from variations in study design and cohort selection (high-risk survivors, cross-sectional and longitudinal studies), follow-up timing (after first pregnancy or after last pregnancy), outcome definitions (chronic hypertension versus CVD events), HDP exposure categorizations (first pregnancy or any recorded pregnancy) and comparator groups (pooling nulliparous women with women without HDP or combining brothers and unaffected sisters).

### Limitations

Our study has several strengths including individual-level linkage of data from population-based Norwegian registries allowing sibling identification, ascertainment of complete pregnancy history to create HDP trajectories, and triangulation of findings using three sibling-based models.

However, our findings have important limitations. We lacked data on non-fatal CVD outcomes and information on important cardiovascular risk factors (such as body mass index, smoking status, and medication use) as these were unavailable or inconsistently reported in earlier periods.

HDP trajectories in affected women were used as proxies for shared familial factors for unaffected siblings. The broad classification into high-risk and low-risk trajectories was chosen to ensure adequate statistical power in sibling-based models. However, additional complications linked to placental dysfunction that could further influence CVD risks were not included, given the scope of the study. Furthermore, information on parental CVD history was also not available.

Findings from sibling-based models may not generalize to all women, since women without siblings, those whose parents could not be identified, those with incomplete pregnancy histories, and those who had not yet reproduced by the end of follow-up were excluded by design. Additionally, findings from sibling-based models may be biased if one sibling’s health outcomes influence the other’s health-seeking behaviors,^29^ potentially delaying CVD mortality or affecting HDP trajectories categorization. Furthermore, misclassification of HDP trajectory could conflate the number of informative family strata, affecting the precision and validity of sibling-comparison model estimates. Therefore, findings may not be transportable to populations with different genetic, socioeconomic contexts and healthcare systems.

### Clinical and research implications

Our findings suggest that CVD risk stratification should account for HDP trajectory patterns and that women with high-risk HDP trajectories should be considered for additional monitoring and prevention strategies to reduce premature CVD mortality. Future studies should investigate longitudinal CVD risk factor development across the reproductive life-course to determine whether low-risk HDP trajectories reflect more favorable post-reproductive cardiometabolic profiles and whether familial aggregation of risk factors differs based on HDP subtype- and trajectory-specific patterns.

## Conclusion

Individual-specific factors predominantly drive the heterogeneity in the HDP-CVD mortality association among women with HDP, while shared familial factors contribute modestly to CVD mortality risk among women without HDP.

## Acknowledgments

A previous version of this work was presented at the Annual Meeting of the Society for Epidemiologic Research in Chicago, Illinois, June 13-16¸ 2022, the Annual Meeting of the Society for Pediatric and Perinatal Epidemiologic Research, Chicago, Illinois, June 13-14, 2022 and the Norwegian Conference on Epidemiology in Tromsø, Norway, October 26-27, 2022.

## Supplementary Material

Web_Material_kwaf257

## Data Availability

Researchers can directly apply for access to data at the Norwegian Institute of Public Health (www.fhi.no/en/hd/access-to-data/about-health-registries) and Statistics Norway (www.ssb.no/en/data-til-forskning). STATA and R codes are available upon request.
