# Supplementary material for: Hypertensive disorders of pregnancy, maternal cardiovascular disease mortality and the role of familial predisposition: a Norwegian population-based sibling-comparison, sibling-spillover and negative-control cohort study
Source: Am J Epidemiol. 2025 Nov 17;195(4):991–1000. doi: 10.1093/aje/kwaf257 (PMC13066338; doi:10.1093/aje/kwaf257)
Supplement: Web_Material_kwaf257 [file web_material_kwaf257.zip › paper1_final_supplementary.docx]

**Supplementary Data**

**Title:** Hypertensive Disorders of Pregnancy, Maternal Cardiovascular Disease Mortality and the role of Familial Predisposition: A Norwegian Population-Based Sibling-Comparison, Sibling-Spillover and Negative-Control Cohort Study

**Authors**: Aditi Singh, Sage Wyatt, Liv Grimstvedt Kvalvik, Rolv Skjærven

**Table of Contents**

[**Figure S1.** Hypertensive disorders of pregnancy (HDP) risk trajectories derived from population-level associations between HDP and cardiovascular disease (CVD) death before age 70. Norway 1967-2020. 2](#_Toc213939929)

[**Figure S2.** Flowchart illustrating the identification of women without children. Norway, 1967-2020. 3](#_Toc213939930)

[**Figure S3.** Cardiovascular disease (CVD) death before age 70 by preeclampsia (PE) and gestational hypertension (GH). Norway 1967-2020. 4](#_Toc213939931)

[**Figure S4.** Distribution plot of bias-corrected hazard ratios (HR) as reported in Supplementary Table S7. 6](#_Toc213939932)

[**Appendix S1.** Description of the probabilistic bias analysis conducted to account for exposure misclassification of mild hypertensive disorders of pregnancy (HDP). 7](#_Toc213939933)

[**Table S1.** Characteristics of women. Norway, 1967-2020. 8](#_Toc213939934)

[**Table S2.** Characteristics women with hypertensive disorders of pregnancy (HDP) according to total number of pregnancies and presence of HDP in first and later pregnancies. Norway, 1967-2020 9](#_Toc213939935)

[**Table S3.** Characteristics of women in the sibling-cohort (sibling-comparison) by hypertensive disorders of pregnancy (HDP) trajectories. Norway 1967-2020. 10](#_Toc213939936)

[**Table S4.** Characteristics of women without hypertensive disorders of pregnancies (HDP) according to their sister’s and sister-in-law’s HDP status. Norway 1967-2020. 11](#_Toc213939937)

[**Table S5.** Direct comparison of hypertensive disorders of pregnancy (HDP) trajectories. Norway, 1967-2020. 12](#_Toc213939938)

[**Table S6.** Adjusted hazard ratios and 95% confidence intervals from sensitivity analyses. Norway, 1967-2020. 13](#_Toc213939939)

[**Table S7.** Bias-corrected hazard ratios (HR) and 95% simulation intervals (SI) from the probabilistic bias analysis accounting for misclassification of mild hypertensive disorders of pregnancy (HDP). Norway, 1967-2020. 14](#_Toc213939940)

**Figure S1.** Hypertensive disorders of pregnancy (HDP) risk trajectories derived from population-level associations between HDP and cardiovascular disease (CVD) death before age 70. Norway 1967-2020.


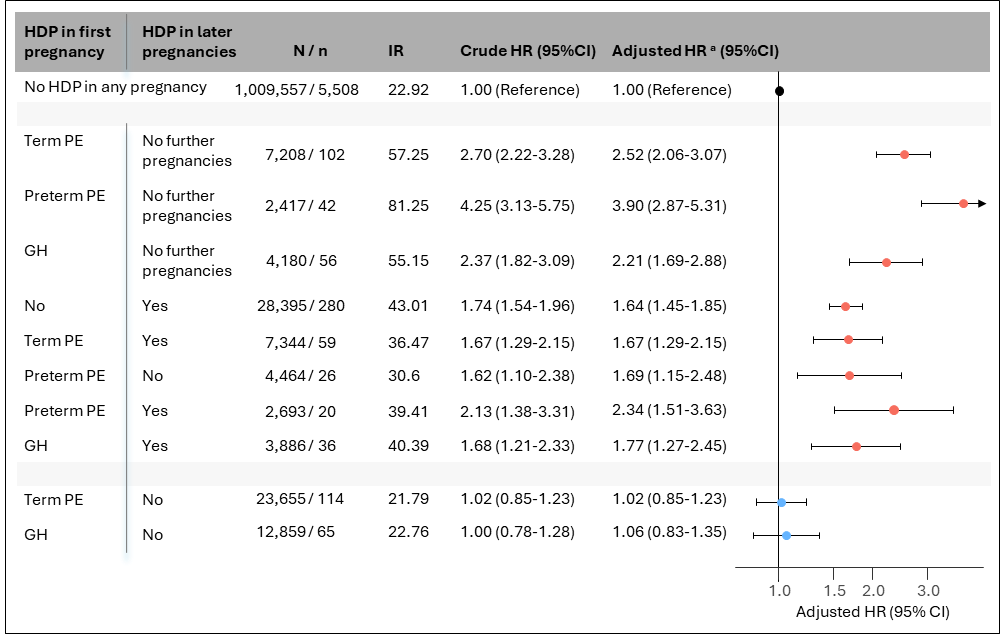


HDP Hypertensive disorders of pregnancy. PE Preeclampsia. GH Gestational hypertension. CVD Cardiovascular disease. IR Incidence rate per 100,000 person-years. HR Hazard ratio. CI Confidence interval.

The estimates for categories labelled in blue constitute ‘low-risk’ trajectories; those labelled red comprise ‘high-risk' trajectories.

a Adjusted for women's year of birth (restricted cubic splines with knots at 1955, 1965 and 1975), country of origin (Norwegian and other), age at first pregnancy (restricted cubic splines with knots at 20, 30 and 40 years), year of first pregnancy (restricted cubic splines with knots at 1975, 1985 and 1999), pre-pregnancy comorbidities (presence of chronic hypertension, chronic kidney disease, epilepsy, rheumatoid arthritis or pregestational diabetes mellitus: yes and no), total number of pregnancies (1, 2 and ≥3), and highest educational attainment (<11, 11-13 and ≥14 years of schooling).

**Figure S2.** Flowchart illustrating the identification of women without children. Norway, 1967-2020.


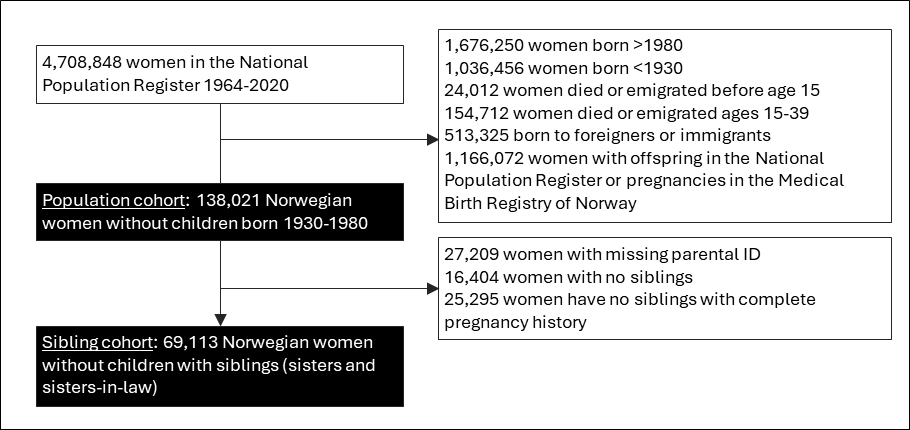


MBRN Medical Birth Registry of Norway. HDP Hypertensive disorders of pregnancy.

Sister-in-law were identified as those who had all their pregnancies with only one partner (full brother of the woman).

**Figure S3.** Cardiovascular disease (CVD) death before age 70 by preeclampsia (PE) and gestational hypertension (GH). Norway 1967-2020.


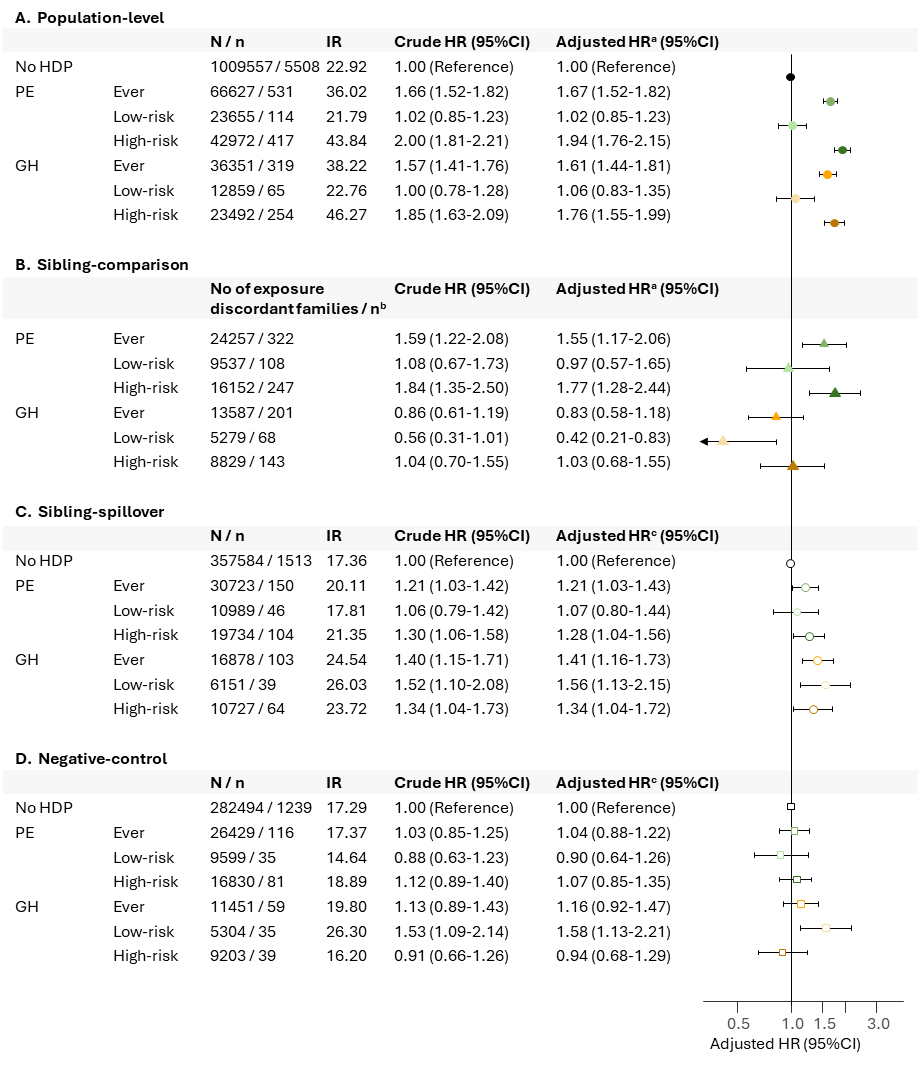


HDP Hypertensive disorders of pregnancy. PE Preeclampsia. GH Gestational hypertension. CVD Cardiovascular disease. IR Incidence rate per 100,000 person-years. HR Hazard ratio. CI Confidence interval.

Women with PE were categorized as low-risk (term PE in first pregnancy followed by no HDPs in subsequent pregnancies) and high-risk (all other PE patterns). Likewise, women with GH were categorized as low-risk (GH in first pregnancy followed by no HDPs) and high-risk (all other GH patterns). Women experiencing PE in the first pregnancy and GH in the second pregnancy and vice versa were included in both high-risk GH and high-risk PE categories.

a Adjusted for women's year of birth (restricted cubic splines with knots at 1955, 1965 and 1975), country of origin (Norwegian and other), age at first pregnancy (restricted cubic splines with knots at 20, 30 and 40 years), year of first pregnancy (restricted cubic splines with knots at 1975, 1985 and 1999), pre-pregnancy comorbidities (presence of chronic hypertension, chronic kidney disease, epilepsy, rheumatoid arthritis or pregestational diabetes mellitus: yes and no), total number of pregnancies (1, 2 and ≥3), and highest educational attainment (<11, 11-13 and ≥14 years of schooling).

b Number of exposure-discordant families where at least one sister developed the outcome (CVD death).

c Adjusted for covariates listed under (a), birth order (first-born and later-born), and number of siblings (1, 2 and ≥3; sisters for sibling-spillover model and sisters-in-law for negative-control model).

**Figure S4.** Distribution plot of bias-corrected hazard ratios (HR) as reported in Supplementary Table S7.


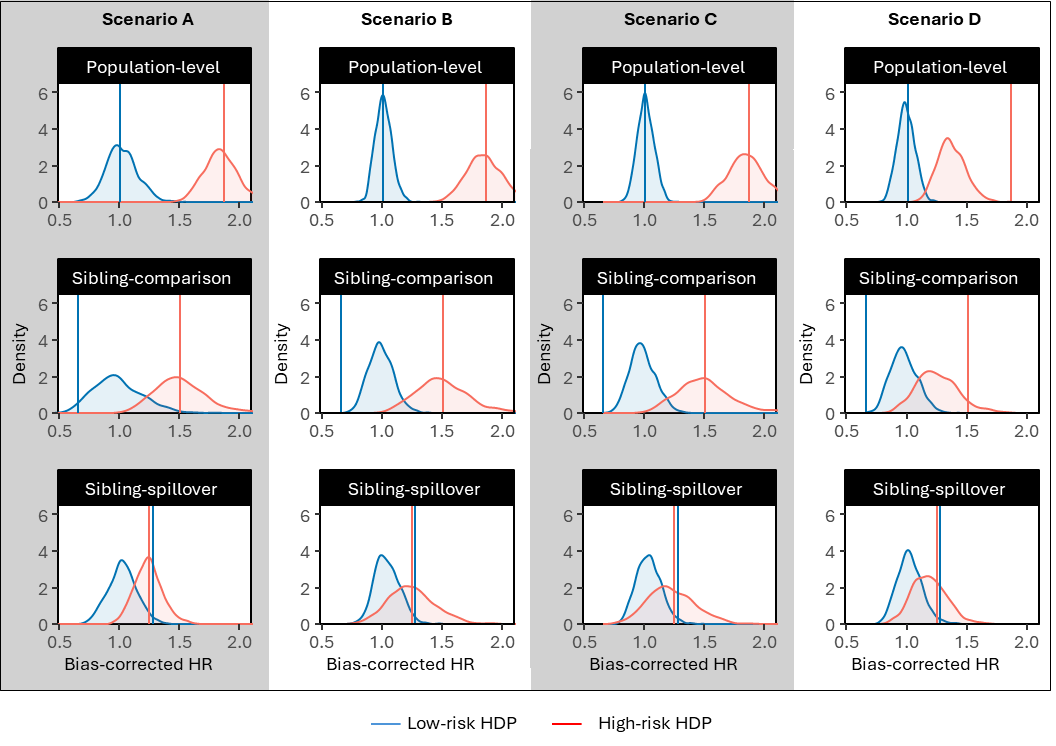


Solid lines represent the adjusted hazard ratios (HRs) based on the sibling-comparison cohort dataset. Density plots represent the distribution of bias-corrected HRs for the three models and four misclassification scenarios.

**Appendix S1.** Description of the probabilistic bias analysis conducted to account for exposure misclassification of mild hypertensive disorders of pregnancy (HDP).

To account for potential exposure misclassification of mild HDP, we conducted a probabilistic bias analysis using Monte Carlo simulation. The code used was adapted from Fox et al (1).

Exposure misclassification was assessed under four scenarios: (scenario A) sampling of sensitivity, specificity and prevalence; (scenario B) direct sampling of positive and negative predictive values; (scenario C) scenario B plus additional misclassification probability if a younger sister had HDP; and (scenario D) scenario B plus reclassification of women with HDP and only one pregnancy as high-risk HDP.

Bias parameters (sensitivity and specificity for Scenario A; positive predictive values [PPV] and negative predictive values [NPV] for Scenario B-D) were based on findings reported by Moth et al. [Table 4 in their paper (2)] and varied by time period (1967-1985 and >1985).

In each of the 1,000 simulation iterations PPV and NPV were used to probabilistically reclassify individuals initially categorized as having no HDP or low-risk HDP, via Bernoulli trials; those with high-risk HDP were not reclassified. For Scenario A, NPV and PPV were calculated using specificity, sensitivity, and prevalence. For Scenario B-D, PPV and NPV were directly sampled.

Bias-corrected hazard ratios (HR) were then estimated for the three models (population-level, sibling-comparison, and sibling-spillover) using the dataset with the ‘correctly classified’ exposure.

Random error was incorporated by taking the natural log of each bias-adjusted HR and subtracting a random value drawn from a standard normal distribution (mean=0, standard deviation=1) multiplied by its standard error and then exponentiating back to HR.

Results were reported as median HRs with 95% simulation intervals (SI; corresponding to the 2.5^th^ and 97.5^th^ percentiles of the bias-corrected HRs) accounting for both systematic and random error.

**References**

1. Fox MP, MacLehose RF, Lash TL. SAS and R code for probabilistic quantitative bias analysis for misclassified binary variables and binary unmeasured confounders. *Int J Epidemiol* 2023;52(5):1624–33.

2. Moth FN, Sebastian TR, Horn J, et al. Validity of a selection of pregnancy complications in the Medical Birth Registry of Norway. *Acta Obstet Gynecol Scand* 2016;95(5):519–27.

**Table S1.** Characteristics of women. Norway, 1967-2020.

|  | Population-cohort | Sibling-cohort | Women with  no siblings | Women with  no children |
| --- | --- | --- | --- | --- |
| Number of women | 1,106,658 | 628,345 | 478,313 | 138,021 |
| Age at first pregnancy (years) |  |  |  |  |
| <20 | 129,238 (11.7) | 81,347 (12.9) | 47,891 (10.0) |  |
| 20-29 | 760,212 (68.7) | 435,014 (69.2) | 325,198 (68.0) |  |
| 30-39 | 208,890 (18.9) | 108,133 (17.2) | 100,757 (21.1) |  |
| ≥40 | 8,318 (0.8) | 3,851 (0.6) | 4,467 (0.9) |  |
| Mean age at first pregnancy (years) | 25.3 ± 5.0 | 25.0 ± 4.9 | 25.6 ± 5.1 |  |
| Mean age at last pregnancy (years) | 30.2 ± 5.1 | 30.3 ± 5.1 | 30.2 ± 5.2 |  |
| Year of first pregnancy |  |  |  |  |
| 1967-1980 | 331,655 (30.0) | 158,317 (25.2) | 173,338 (36.2) |  |
| 1981-1999 | 433,018 (39.1) | 309,128 (49.2) | 123,890 (25.9) |  |
| 2000-2020 | 341,985 (30.9) | 160,900 (25.6) | 181,085 (37.9) |  |
| Woman’s year of birth |  |  |  |  |
| <1955 | 286,674 (25.9) | 118,142 (18.8) | 168,532 (35.2) | 56,437 (40.9) |
| 1955-1964 | 260,506 (23.5) | 196,775 (31.3) | 63,731 (13.3) | 31,173 (22.6) |
| 1965-1974 | 277,813 (25.1) | 189,223 (30.1) | 88,590 (18.5) | 32,098 (23.3) |
| ≥1975 | 281,665 (25.5) | 124,205 (19.8) | 157,460 (32.9) | 18,313 (13.3) |
| Woman’s country of origin |  |  |  |  |
| Norway | 964,123 (87.1) | 597,985 (95.2) | 366,138 (76.5) | 138,021 (100) |
| Other | 142,462 (12.9) | 30,360 (4.8) | 112,102 (23.4) | 0 |
| Missing | 73 (<0.01) | 0 | 73 (<0.01) | 0 |
| Woman’s educational attainment (years) |  |  |  |  |
| <11 | 211,105 (19.1) | 113,358 (18.0) | 97,747 (20.4) | 33,011 (24.5) |
| 11-13 | 420,023 (38.0) | 242,061 (38.5) | 177,962 (37.2) | 48,771 (36.2) |
| ≥14 | 464,169 (41.9) | 271,828 (43.3) | 192,341 (40.2) | 52,867 (39.3) |
| Missing | 11,361 (1.0) | 1,098 (0.2) | 10,263 (2.1) | 0 |
| Pre-pregnancy comorbidities ^a^ | 20,873 (1.9) | 12,426 (2.0) | 8,447 (1.8) |  |
| Total number of pregnancies |  |  |  |  |
| 1 | 191,271 (17.3) | 92,517 (14.7) | 98,754 (20.6) |  |
| 2 | 544,514 (49.2) | 306,103 (48.7) | 238,411 (49.8) |  |
| 3 | 287,753 (26.0) | 178,377 (28.4) | 109,376 (22.9) |  |
| ≥4 | 83,120 (7.5) | 51,348 (8.2) | 31,772 (6.6) |  |
| Emigration | 24,481 (2.2) | 4,983 (0.8) | 19,498 (4.1) | 1,885 (1.4) |
| Median follow-up after last pregnancy (years) | 24 (12-35) | 24 (15-33) | 22 (9-37) | 19 (9-30) |
| Median age at end of follow-up (years) | 54 (44-65) | 55 (47-63) | 53 (41-69) | 59 (49-70) |
| Parental identification number | 909,605 (82.2) | 628,345 (100.0) | 281,260 (58.8) | 110,812 (80.3) |
| Woman’s birth order |  |  |  |  |
| First | 402,048 (44.2) | 212,795 (33.9) | 189,253 (67.3) | 52,071 (47.0) |
| Later | 507,557 (55.8) | 415,550 (66.1) | 92,007 (32.7) | 58,741 (53.0) |
| CVD deaths before age 70 | 6,308 (0.6) | 2,796 (0.4) | 3,512 (0.7) | 2,506 (1.8) |
| Death from non-CVD causes before age 70 | 40,901 (3.7) | 19,831 (3.2) | 21,070 (4.4) | 12,041 (8.7) |

a Presence of chronic kidney disease, rheumatoid arthritis, epilepsy, chronic hypertension and diabetes mellites before first pregnancy

**Table S2.** Characteristics women with hypertensive disorders of pregnancy (HDP) according to total number of pregnancies and presence of HDP in first and later pregnancies. Norway, 1967-2020

| HDP trajectories | No HDP | Low-risk HDP | | High-risk HDP | | | | | | | |
| --- | --- | --- | --- | --- | --- | --- | --- | --- | --- | --- | --- |
| Total number of pregnancies | ≥1 | ≥2 | ≥2 | 1 | 1 | 1 | ≥2 | ≥2 | ≥2 | ≥2 | ≥2 |
| HDP in first pregnancy | No HDP | Term PE | GH | Term PE | Preterm PE | GH | No HDP | Term PE | Preterm PE | Preterm PE | GH |
| HDP in later pregnancies | No | No | No | No additional pregnancies | | | Yes | Yes | No | Yes | Yes |
| Number of women | 1,009,557 | 23,655 | 12,859 | 7,208 | 2,417 | 4,180 | 28,395 | 7,344 | 4,464 | 2,693 | 3,886 |
| Age at first  pregnancy (years) ^a^ | 25.2 ± 5.0 | 24.8 ± 4.6 | 25.6 ± 4.7 | 28.5 ± 6.2 | 29.8 ± 6.3 | 29.7 ± 6.4 | 24.3 ± 4.5 | 25.1 ± 4.6 | 25.4 ± 4.8 | 25.8 ± 4.7 | 25.7 ± 4.6 |
| Year of first birth | | | | | | | | | | | |
| 1967-1980 | 305,668 (30.3) | 5,660 (23.9) | 3,665 (28.5) | 1,600 (22.2) | 319 (13.2) | 1,157 (27.7) | 9,602 (33.8) | 1,752 (23.9) | 690 (15.5) | 360 (13.4) | 1,181 (30.4) |
| 1981-1999 | 394,137 (39.0) | 10,045 (42.5) | 4,287 (33.3) | 2,821 (39.1) | 934 (38.6) | 1,246 (29.8) | 11,703 (41.2) | 3,360 (45.8) | 1,800 (40.3) | 1,227 (45.6) | 1,456 (37.5) |
| 2000-2020 | 309,752 (30.7) | 7,950 (33.6) | 4,907 (38.2) | 2,787 (38.7) | 1,164 (48.2) | 1,777 (42.5) | 7,090 (25.0) | 2,232 (30.4) | 1,974 (44.2) | 1,106 (41.1) | 1,249 (32.1) |
| Woman’s country of origin ^b^ | | | | | | | | | | | |
| Norway | 877,091 (86.9) | 21,418 (90.5) | 11,698 (91.0) | 6,174 (85.7) | 1,999 (82.7) | 3,570 (85.4) | 25,564 (90.0) | 6,746 (91.9) | 3,891 (87.2) | 2,362 (87.7) | 3,611 (92.9) |
| Other | 132,398 (13.1) | 2,235 (9.4) | 1,161 (9.0) | 1,034 (14.3) | 417 (17.3) | 610 (14.6) | 2,831 (10.0) | 598 (8.1) | 573 (12.8) | 331 (12.3) | 275 (7.1) |
| Woman’s year of birth | | | | | | | | | | | |
| <1955 | 264,200 (26.2) | 4,754 (20.1) | 2,949 (22.9) | 1,795 (24.9) | 429 (17.7) | 1,249 (29.9) | 7,901 (27.8) | 1,489 (20.3) | 605 (13.6) | 322 (12.0) | 979 (25.2) |
| 1955-1964 | 237,876 (23.6) | 5,409 (22.9) | 3,139 (24.4) | 1,681 (23.3) | 500 (20.7) | 922 (22.1) | 6,745 (23.8) | 1,844 (25.1) | 805 (18.0) | 542 (20.1) | 1,043 (26.8) |
| 1965-1974 | 251,253 (24.9) | 6,763 (28.6) | 2,889 (22.5) | 2,004 (27.8) | 842 (34.8) | 1,028 (24.6) | 7,555 (26.6) | 2,165 (29.5) | 1,435 (32.1) | 960 (35.6) | 920 (23.7) |
| ≥1975 | 256,228 (25.4) | 6,729 (28.4) | 3,882 (30.2) | 1,728 (24.0) | 646 (26.7) | 981 (23.5) | 6,194 (21.8) | 1,846 (25.1) | 1,619 (36.3) | 869 (32.3) | 944 (24.3) |
| Woman’s educational attainment (years) | | | | | | | | | | | |
| <11 | 192,850 (19.1) | 4,358 (18.4) | 1,939 (15.1) | 1,585 (22.0) | 502 (20.8) | 872 (20.9) | 5,876 (20.7) | 1,280 (17.4) | 776 (17.4) | 465 (17.3) | 601 (15.5) |
| 11-13 | 382,436 (37.9) | 9,177 (38.8) | 4,745 (36.9) | 2,916 (40.5) | 955 (39.5) | 1,560 (37.3) | 11,330 (39.9) | 2,872 (39.1) | 1,594 (35.7) | 950 (35.3) | 1,488 (38.3) |
| ≥14 | 423,534 (42.0) | 10,024 (42.4) | 6,120 (47.6) | 2,592 (36.0) | 912 (37.7) | 1,659 (39.7) | 11,060 (39.0) | 3,162 (43.1) | 2,061 (46.2) | 1,263 (46.9) | 1,785 (45.9) |
| Missing | 10,737 (1.1) | 96 (0.4) | 55 (0.4) | 115 (1.6) | 48 (2.0) | 89 (2.1) | 129 (0.5) | 30 (0.4) | 33 (0.7) | 15 (0.6) | 12 (0.3) |
| Pre-pregnancy  comorbidities ^c^ | 17,187 (1.7) | 707 (3.0) | 227 (1.8) | 397 (5.5) | 340 (14.1) | 149 (3.6) | 955 (3.4) | 361 (4.9) | 230 (5.2) | 227 (8.4) | 93 (2.4) |
| Emigration | 23,113 (2.3) | 245 (1.0) | 129 (1.0) | 305 (4.2) | 100 (4.1) | 193 (4.6) | 231 (0.8) | 55 (0.7) | 52 (1.2) | 37 (1.4) | 21 (0.5) |

HDP Hypertensive disorders of pregnancy. PE Preeclampsia. GH Gestational hypertension.

a Mean and standard deviation

b Country of origin missing for n=72 (<0.01)

c Presence of chronic kidney disease, rheumatoid arthritis, epilepsy, chronic hypertension and diabetes mellites before first pregnancy

**Table S3.** Characteristics of women in the sibling-cohort (sibling-comparison) by hypertensive disorders of pregnancy (HDP) trajectories. Norway 1967-2020.

| HDP trajectories | No HDP | Low-risk HDP | High-risk HDP |
| --- | --- | --- | --- |
| Number of women | 401,623 | 15,292 | 24,224 |
| Age at first pregnancy (years) | | | |
| <20 | 55,988 (13.9) | 1,892 (12.4) | 3,077 (12.7) |
| 20-29 | 277,428 (69.1) | 10,970 (71.7) | 16,433 (67.8) |
| 30-39 | 65,894 (16.4) | 2,402 (15.7) | 4,456 (18.4) |
| ≥40 | 2,313 (0.6) | 28 (0.2) | 258 (1.1) |
| Year of first pregnancy | | | |
| 1967-1980 | 101,422 (25.3) | 3290 (21.5) | 5481 (22.6) |
| 1981-1999 | 201,271 (50.1) | 7541 (49.3) | 12247 (50.6) |
| 2000-2020 | 98,930 (24.6) | 4461 (29.2) | 6496 (26.8) |
| Woman’s year of birth | | | |
| <1955 | 73,165 (18.2) | 2,253 (14.7) | 4,084 (16.9) |
| 1955-1964 | 130,055 (32.4) | 4,777 (31.2) | 7,475 (30.9) |
| 1965-1974 | 120,842 (30.1) | 4,785 (31.3) | 7,915 (32.7) |
| ≥1975 | 77,561 (19.3) | 3,477 (22.7) | 4,750 (19.6) |
| Woman’s country of origin | | | |
| Norway | 381,300 (94.9) | 14,640 (95.7) | 23,118 (95.4) |
| Other | 20,323 (5.1) | 652 (4.3) | 1,106 (4.6) |
| Woman’s educational attainment (years) | | | |
| <11 | 76,065 (18.9) | 2,662 (17.4) | 4,879 (20.1) |
| 11-13 | 155,230 (38.7) | 5,929 (38.8) | 9,538 (39.4) |
| ≥14 | 169,527 (42.2) | 6,682 (43.7) | 9,768 (40.3) |
| Missing | 801 (0.2) | 19 (0.1) | 39 (0.2) |
| Pre-pregnancy comorbidities ^a^ | 7,119 (1.8) | 402 (2.6) | 1,113 (4.6) |
| Total number of pregnancies | | | |
| 1 | 59,486 (14.8) | 0 | 4,684 (19.3) |
| 2 | 193,599 (48.2) | 9,217 (60.3) | 8.947 (36.9) |
| 3 | 114,784 (28.6) | 4,777 (31.2) | 7,464 (30.8) |
| ≥4 | 33,754 (8.4) | 1,298 (8.5) | 3,129 (12.9) |
| Emigration | 3,347 (0.8) | 78 (0.5) | 150 (0.6) |
| Woman’s birth order | | | |
| First | 130,253 (32.4) | 5,154 (33.7) | 8,278 (34.2) |
| Later | 271,370 (67.6) | 10,138 (66.3) | 15,946 (65.8) |

HDP Hypertensive disorders of pregnancy.

a Presence of chronic kidney disease, rheumatoid arthritis, epilepsy, chronic hypertension and diabetes mellites before first pregnancy

**Table S4.** Characteristics of women without hypertensive disorders of pregnancies (HDP) according to their sister’s and sister-in-law’s HDP status. Norway 1967-2020.

|  | Sisters | | | Sisters-in-law | | |
| --- | --- | --- | --- | --- | --- | --- |
| Sibling’s HDP status | No HDP | Low-risk HDP ^a^ | High-risk HDP ^b^ | No HDP | Low-risk HDP ^a^ | High-risk HDP ^b^ |
| Number of women without HDP | 357,584 | 16,711 | 27,328 | 282,494 | 14,618 | 23,262 |
| Age at first pregnancy (years) | | | | | | |
| <20 | 49,576 (13.9) | 2,293 (13.7) | 4,119 (15.1) | 37,652 (13.3) | 1,938 (13.3) | 3,423 (14.7) |
| 20-29 | 246,679 (69.0) | 11,740 (70.3) | 19,009 (69.6) | 195,699 (69.3) | 10,182 (69.7) | 16,121 (69.3) |
| 30-39 | 59,259 (16.6) | 2,596 (15.5) | 4,039 (14.8) | 47,409 (16.8) | 2,407 (16.5) | 3,570 (15.3) |
| ≥40 | 2,070 (0.6) | 82 (0.5) | 161 (0.6) | 1,734 (0.6) | 91 (0.6) | 148 (0.6) |
| Year of first pregnancy | | | | | | |
| 1967-1980 | 90,514 (25.3) | 3,997 (23.9) | 6,911 (25.3) | 79,249 (28.1) | 3,891 (26.6) | 6,653 (28.6) |
| 1981-1999 | 178,441 (49.9) | 8,537 (51.1) | 14,293 (52.3) | 142,088 (50.3) | 7,453 (51.0) | 12,114 (52.1) |
| 2000-2020 | 88,629 (24.8) | 4,177 (25.0) | 6,124 (22.4) | 61,157 (21.6) | 3,274 (22.4) | 4,495 (19.3) |
| Woman’s year of birth | | | | | | |
| <1955 | 65,758 (18.4) | 2,730 (16.3) | 4,677 (17.1) | 58,857 (20.8) | 2,776 (19.0) | 4,787 (20.6) |
| 1955-1964 | 115,328 (32.3) | 5,426 (32.5) | 9,301 (34.0) | 96,527 (34.2) | 5,057 (34.6) | 8,273 (35.6) |
| 1965-1974 | 107,134 (30.0) | 5,215 (31.2) | 8,493 (31.1) | 81,755 (28.9) | 4,339 (29.7) | 6,947 (29.9) |
| ≥1975 | 69,364 (19.4) | 3,340 (20.0) | 4,857 (17.8) | 45,355 (16.1) | 2,446 (16.7) | 3,255 (14.0) |
| Woman’s country of origin | | | | | | |
| Norway | 339,211 (94.9) | 16,001 (95.8) | 26,088 (95.5) | 270,108 (95.6) | 14,097 (96.4) | 22,535 (96.9) |
| Other | 18,373 (5.1) | 710 (4.2) | 1,240 (4.5) | 12,386 (4.4) | 521 (3.6) | 727 (3.1) |
| Woman’s educational attainment (years) | | | | | | |
| <11 | 67,165 (18.8) | 3,196 (19.1) | 5,704 (20.9) | 51,365 (18.2) | 2,643 (18.1) | 4,464 (19.2) |
| 11-13 | 137,971 (38.6) | 6,504 (38.9) | 10,755 (39.4) | 109,793 (38.9) | 5,698 (39.0) | 9,443 (40.6) |
| ≥14 | 151,729 (42.4) | 6,986 (41.8) | 10,812 (39.6) | 120,850 (42.8) | 6,253 (42.8) | 9,327 (40.1) |
| Missing | 719 (0.2) | 25 (0.1) | 57 (0.2) | 486 (0.2) | 24 (0.2) | 28 (0.1) |
| Pre-pregnancy comorbidities ^c^ | 6,244 (1.7) | 316 (1.9) | 559 (2.0) | 5,022 (1.8) | 286 (2.0) | 392 (1.7) |
| Total number of pregnancies | | | | | | |
| 1 | 53,162 (14.9) | 2,315 (13.9) | 4,009 (14.7) | 40,972 (14.5) | 2,062 (14.1) | 3,240 (13.9) |
| 2 | 172,910 (48.4) | 7,953 (47.6) | 12,736 (46.6) | 135,564 (48.0) | 6,701 (45.8) | 10,552 (45.4) |
| 3 | 101,860 (28.5) | 4,942 (29.6) | 7,982 (29.2) | 82,155 (29.1) | 4,511 (30.9) | 7,044 (30.3) |
| ≥4 | 29,652 (8.3) | 1,501 (9.0) | 2,601 (9.5) | 23,803 (8.4) | 1,344 (9.2) | 2,426 (10.4) |
| Woman’s birth order | | | | | | |
| First | 117,291 (32.8) | 5,061 (30.3) | 7,901 (28.9) | 88,510 (31.3) | 4,299 (29.4) | 6,546 (28.1) |
| Later | 240,293 (67.2) | 11,650 (69.7) | 19,427 (71.1) | 193,984 (68.7) | 10,319 (70.6) | 16,716 (71.9) |
| Emigration | 2,992 (0.8) | 131 (0.8) | 224 (0.8) | 2,168 (0.8) | 108 (0.7) | 158 (0.7) |
| Number of sisters | | | | | | |
| 0 |  |  |  | 15,0975 (53.4) | 7,575 (51.8) | 11,718 (50.4) |
| 1 | 255,818 (71.5) | 9,131 (54.6) | 14,103 (51.6) | 89,954 (31.8) | 4,664 (31.9) | 7,453 (32) |
| 2 | 78,027 (21.8) | 5,152 (30.8) | 8,850 (32.4) | 29,941 (10.6) | 1,678 (11.5) | 2,794 (12) |
| ≥3 | 23,739 (6.6) | 2,428 (14.5) | 4,375 (16.0) | 11,624 (4.1) | 701 (4.8) | 1,297 (5.6) |
| Number of sisters-in-law | | | | | | |
| 0 | 224,012 (62.6) | 10,417 (62.3) | 17,088 (62.5) |  |  |  |
| 1 | 96,893 (27.1) | 4,470 (26.7) | 7,242 (26.5) | 215,268 (76.2) | 8,835 (60.4) | 13,061 (56.1) |
| 2 | 27,579 (7.7) | 1,358 (8.1) | 2,123 (7.8) | 53,239 (18.8) | 4,102 (28.1) | 6,895 (29.6) |
| ≥3 | 9,100 (2.5) | 466 (2.8) | 875 (3.2) | 13,987 (5.0) | 1,681 (11.5) | 3,306 (14.2) |

¨

HDP Hypertensive disorders of pregnancy.

a All siblings with low-risk HDP trajectory.

b At least one sibling with a high-risk HDP trajectory.

c Presence of chronic kidney disease, rheumatoid arthritis, epilepsy, chronic hypertension or diabetes mellites before first pregnancy

**Table S5.** Direct comparison of hypertensive disorders of pregnancy (HDP) trajectories. Norway, 1967-2020.

|  | N | n | IR | Crude HR (95% CI) | Adjusted HR (95% CI) ^a^ | Crude HR (95% CI) | Adjusted HR (95% CI) ^a^ | Crude HR  (95% CI) | Adjusted HR (95% CI) ^a^ |
| --- | --- | --- | --- | --- | --- | --- | --- | --- | --- |
| Women from families with no HDP | 357,584 | 1,513 | 17.36 | 1.00 (Reference) | 1.00 (Reference) |  |  |  |  |
| Women without HDP whose sisters had low-risk HDP | 16,711 | 85 | 21.15 | 1.26 (1.01-1.56) | 1.28 (1.03-1.60) | 1.24 (0.91-1.67) | 1.27 (0.89-1.78) |  |  |
| Women with low-risk HDP | 15,292 | 60 | 17.15 | 1.04 (0.80-1.35) | 1.01 (0.78-1.30) | 1.00 (Reference) | 1.00 (Reference) |  |  |
| Women without HDP whose sisters had high-risk HDP | 27,328 | 146 | 21.70 | 1.28 (1.08-1.52) | 1.25 (1.06-1.49) |  |  | 0.64 (0.52-0.79) | 0.67 (0.53-0.84) |
| Women with high-risk HDP | 24,224 | 192 | 34.69 | 2.01 (1.73-2.34) | 1.87 (1.61-2.17) |  |  | 1.00 (Reference) | 1.00 (Reference) |

HDP Hypertensive disorders of pregnancy. PE Preeclampsia. GH Gestational hypertension. CVD Cardiovascular disease. IR Incidence rate per 100,000 person-years. HR Hazard ratio. CI Confidence interval.

a Adjusted for women's year of birth (restricted cubic splines with knots at 1955, 1965 and 1975), country of origin (Norwegian and other), age at first pregnancy (restricted cubic splines with knots at 20, 30 and 40 years), year of first pregnancy (restricted cubic splines with knots at 1975, 1985 and 1999), pre-pregnancy comorbidities (presence of chronic hypertension, chronic kidney disease, epilepsy, rheumatoid arthritis or pregestational diabetes mellitus: yes and no), total number of pregnancies (1, 2 and ≥3), and highest educational attainment (<11, 11-13 and ≥14 years of schooling).

**Table S6.** Adjusted hazard ratios and 95% confidence intervals from sensitivity analyses. Norway, 1967-2020.

|  | **Population-level model:** Women with HDP versus women without HDP | **Sibling-comparison model:** Sisters with HDP versus sisters without HDP | **Sibling-spillover model:** Women without HDP—by sister’s HDP status (sisters with HDP versus sisters without HDP) | **Negative-control model:** Women without HDP—by sister-in-law’s HDP status (sisters-in-law with HDP versus sisters-in-law without HDP) |
| --- | --- | --- | --- | --- |
| **Main:** CVD death before age 70 ^a, b^ | | | | |
| Number of women | 1,106,658 | 441,139 | 401,623 | 320,374 |
| HDP in any pregnancy | 1.64 (1.52-1.76) | 1.21 (0.96-1.52) | 1.27 (1.10-1.46) | 1.04 (0.88-1.22) |
| Low-risk HDP | 1.03 (0.89-1.20) | 0.66 (0.44-1.01) | 1.28 (1.03-1.60) | 1.10 (0.85-1.40) |
| High-risk HDP | 1.89 (1.74-2.06) | 1.51 (1.16-1.97) | 1.25 (1.06-1.49) | 1.01 (0.83-1.22) |
| **Sensitivity:** CVD death after 70 years ^a, b, c^ | | | | |
| Number of women | 138,043 | 8,574 | 21,601 | 20,005 |
| HDP in any pregnancy | 1.34 (1.16-1.55) | 1.24 (0.11-13.91) | 1.00 (0.55-1.81) | 0.92 (0.49-1.72) |
| Low-risk HDP | 1.05 (0.77-1.43) | DNC | 0.47 (0.12-1.91) | 0.84 (0.31-2.29) |
| High-risk HDP | 1.45 (1.23-1.70) | 0.64 (0.04-10.74) | 1.29 (0.67-2.46) | 0.97 (0.45-2.10) |
| **Sensitivity:** Death from non-CVD before age 70 ^a, b, d^ | | | | |
| Number of women | 1,106,658 | 441,139 | 401,623 | 320,374 |
| HDP in any pregnancy | 0.98 (0.95-1.02) | 1.05 (0.95-1.15) | 0.96 (0.91-1.02) | 0.98 (0.92-1.04) |
| Low-risk HDP | 0.81 (0.76-0.86) | 0.87 (0.74-1.03) | 0.93 (0.85-1.02) | 1.00 (0.91-1.10) |
| High-risk HDP | 1.03 (0.99-1.08) | 1.12 (0.99-1.26) | 0.98 (0.91-1.05) | 0.97 (0.90-1.05) |
| **Sensitivity:** CVD death between 40-70 among Norwegian women born 1930-1980 ^a^ | | | | |
| Number of women | 832,108 | 375,508 | 341,900 | 280,012 |
| HDP in any pregnancy | 1.55 (1.43-1.68) | 1.25 (0.97-1.61) | 1.22 (1.05-1.42) | 1.10 (0.93-1.30) |
| Low-risk HDP | 1.01 (0.86-1.19) | 0.67 (0.42-1.06) | 1.25 (0.99-1.58) | 1.16 (0.89-1.49) |
| High-risk HDP | 1.79 (1.63-1.96) | 1.61 (1.20-2.15) | 1.20 (1.00-1.44) | 1.07 (0.87-1.32) |
| **Sensitivity:** CVD death between 40-70 among Norwegian women born 1930-1980, including women without children ^a^ | | | | |
| Number of women | 970,129 | 444,958 | 388,545 | 318,545 |
| HDP in any pregnancy | 1.54 (1.42-1.67) | 1.28 (1.01-1.63) | 1.18 (1.03-1.35) | 1.02 (0.88-1.19) |
| Low-risk HDP | 1.02 (0.87-1.20) | 0.80 (0.53-1.20) | 1.27 (1.03-1.56) | 1.07 (0.85-1.35) |
| High-risk HDP | 1.83 (1.67-2.00) | 1.61 (1.22-2.13) | 1.12 (0.95-1.33) | 0.99 (0.82-1.20) |
| Women without children | 2.40 (2.28-2.52) |  |  |  |
| **Sensitivity:** CVD death before age 70, based on the sibling matched-cohort, all sets ^a, b, c, e^ | | | | |
| Number of women |  | 81,082 |  |  |
| HDP in any pregnancy |  | 1.24 (1.02-1.50) |  |  |
| Low-risk HDP |  | 0.74 (0.52-1.05) |  |  |
| High-risk HDP |  | 1.47 (1.19-1.83) |  |  |
| **Sensitivity:** CVD death before age 70, with exposure based on siblings born within seven years of each other ^a, b^ | | | | |
| Number of women |  | 64,842 | 392,025 | 308,610 |
| HDP in any pregnancy |  | 1.18 (0.97-1.44) | 1.23 (1.05-1.45) | 1.09 (0.89-1.31) |
| Low-risk HDP |  | 0.66 (0.46-0.97) | 1.40 (1.10-1.78) | 1.18 (0.88-1.59) |
| High-risk HDP |  | 1.44 (1.15-1.81) | 1.13 (0.92-1.39) | 1.02 (0.79-1.30) |
| **Sensitivity:** CVD death before age 70 among women with no pregnancy complications ^a, b^ | | | | |
| Number of women |  | 334,767 | 295,251 | 235,854 |
| HDP in any pregnancy |  | 1.27 (0.96-1.69) | 1.23 (1.04-1.47) | 0.97 (0.79-1.20) |
| Low-risk HDP |  | 0.82 (0.53-1.29) | 1.28 (0.98-1.68) | 0.83 (0.59-1.18) |
| High-risk HDP |  | 1.56 (1.13-2.17) | 1.21 (0.97-1.50) | 1.05 (0.82-1.35) |

HDP Hypertensive disorders of pregnancy. CVD Cardiovascular disease.

a Adjusted for women's year of birth (restricted cubic splines with knots at 1955, 1965 and 1975), country of origin (Norwegian and other), highest educational attainment (<11, 11-13 and ≥14 years of schooling), total number of pregnancies (0, 1, 2 and ≥3), birth order (first-born and later-born), and number of sisters-in-law (1, 2 and ≥3).

b Adjusted for age at first pregnancy (restricted cubic splines with knots at 20, 30 and 40 years), year of first pregnancy (restricted cubic splines with knots at 1975, 1985 and 1999) and pre-pregnancy comorbidities (presence of chronic hypertension, chronic kidney disease, epilepsy, rheumatoid arthritis or pregestational diabetes mellitus: yes and no).

**Table S7.** Bias-corrected hazard ratios (HR) and 95% simulation intervals (SI) from the probabilistic bias analysis accounting for misclassification of mild hypertensive disorders of pregnancy (HDP). Norway, 1967-2020.

|  |  |  | Scenario A ^a^ | | Scenario B ^b^ | | Scenario C ^b, c^ | | Scenario D ^b, d^ | |
| --- | --- | --- | --- | --- | --- | --- | --- | --- | --- | --- |
| Model | HDP status | Adjusted HR (95% CI)  from Figure 4 | Bias-corrected HR  (95% SI) | Bias (%) ^e^ | Bias-corrected  HR (95% SI) | Bias (%) ^e^ | Bias-corrected  HR (95% SI) | Bias (%) ^e^ | Bias-corrected  HR (95% SI) | Bias (%) ^e^ |
| Population-level | Low-risk HDP | 1.01 (0.78-1.30) | 1.01 (0.78-1.29) | 0 | 1.01 (0.90-1.16) | 0 | 1.01 (0.89-1.15) | 0 | 0.99 (0.86-1.14) | 2 |
|  | High-risk HDP | 1.87 (1.61-2.17) | 1.84 (1.59-2.15) | 2 | 1.85 (1.57-2.19) | 1 | 1.85 (1.58-2.19) | 1 | 1.36 (1.17-1.61) | 38 |
| Sibling-comparison | Low-risk HDP | 0.66 (0.44-1.01) | 0.97 (0.65-1.41) | -32 | 0.99 (0.81-1.21) | -33 | 0.98 (0.80-1.21) | -33 | 0.97 (0.78-1.21) | -32 |
|  | High-risk HDP | 1.51 (1.16-1.97) | 1.50 (1.14-1.98) | 1 | 1.49 (1.12-1.99) | 2 | 1.49 (1.12-2.04) | 2 | 1.24 (0.95-1.65) | 22 |
| Sibling-spillover | Low-risk HDP | 1.28 (1.03-1.60) | 1.03 (0.80-1.27) | 25 | 1.03 (0.86-1.26) | 24 | 1.04 (0.86-1.26) | 24 | 1.02 (0.84-1.25) | 26 |
|  | High-risk HDP | 1.25 (1.06-1.49) | 1.24 (1.03-1.49) | 1 | 1.24 (0.90-1.65) | 1 | 1.21 (0.89-1.63) | 3 | 1.18 (0.92-1.50) | 6 |

HDP Hypertensive disorders of pregnancy. HR Hazard Ratio. SI Simulation Intervals derived from 2.5^th^ and 97.5^th^ percentiles.

a Based on sampled sensitivity and specificity from triangular distributions, varying by year of first pregnancy: for 1967-1985, PPV (min=0.78, max=0.93, mode=0.87) and NPV (min=0.40, max=0.57, mode=0.49); for >1985, PPV (min=0.74, max=0.97, mode=0.90) and NPV (min=0.72, max=0.86, mode=0.80). Prevalence sampled from uniform distribution (min=0.05, max=0.08)

b Based on sampled positive (PPV) and negative predictive values (NPV) from triangular distributions, varying by year of first pregnancy: for 1967-1985, PPV (min=0.78, max=0.93, mode=0.87) and NPV (min=0.40, max=0.57, mode=0.49); for >1985, PPV (min=0.74, max=0.97, mode=0.90) and NPV (min=0.72, max=0.86, mode=0.80).

c Additionally incorporating increased probability of misclassification if the younger sister had HDP

d Reclassification of ‘correctly classified’ women with HDP who had only one pregnancy as ‘high-risk HDP’.

e Bias (%) calculated as (observed HR – bias-corrected HR) / bias-corrected HR.
