# Supplementary material for: Hypertensive disorders of pregnancy, maternal cardiovascular disease mortality and the role of familial predisposition: a Norwegian population-based sibling-comparison, sibling-spillover and negative-control cohort study
Source: Am J Epidemiol. 2025 Nov 17;195(4):991–1000. doi: 10.1093/aje/kwaf257 (PMC13066338; doi:10.1093/aje/kwaf257)
Supplement: Web_Material_kwaf257 [file web_material_kwaf257.zip › paper1_final_supplementary_reference_list.docx]

1. Fox MP, MacLehose RF, Lash TL. SAS and R code for probabilistic quantitative bias analysis for misclassified binary variables and binary unmeasured confounders. *Int J Epidemiol* 2023;52(5):1624–33.

2. Moth FN, Sebastian TR, Horn J, et al. Validity of a selection of pregnancy complications in the Medical Birth Registry of Norway. *Acta Obstet Gynecol Scand* 2016;95(5):519–27.
